# Hide and seek: *de novo* identification in sugar beet reveals impact of non-autonomous LTR retrotransposons

**DOI:** 10.1186/s13100-026-00416-w

**Published:** 2026-09-11

**Authors:** Sophie Maiwald, Ferdinand Maiwald, Tony Heitkam

**Affiliations:** 1 Chair of Molecular Botany, Faculty of Mathematics and Natural Science, Rheinisch- Westfälische Technische Hochschule Aachen, Aachen, 52056 Germany; 2 Chair of Optical 3D-Metrology,Faculty of Environmental Sciences, Technische Universität Dresden, Dresden, 01069 Germany

**Keywords:** Transposable elements, LTR retrotransposons, Non-autonomous, TRIMs, Transposable element identification, Long terminal repeats

## Abstract

Plant genomes are filled with retrotransposons and their derivatives, constantly undergoing sequence diversification and structural rearrangement. Among them, short, non-autonomous retrotransposons lack full coding capacity and often form subfamilies. As a result, non-autonomous retrotransposons are incompletely identified in most to all genome assemblies.

Here, we capitalize on our comprehensive understanding of the transposable element (TE) landscape in sugar beet (*Beta vulgaris*) to assess the extent of the blind spot for non-autonomous long terminal repeat (LTR) retrotransposons. This use case serves to answer if all of these sequences are derivatives of easier-to-identify full-length elements or if there is more variability that is currently overlooked.

For this we applied a semi-automated structural discovery workflow followed by in-depth manual verification to characterize non-autonomous LTR retrotransposons in sugar beet. We retrieve more than 100 non-autonomous LTR retrotransposon families that lack complete autonomous coding capacity, including canonical terminal-repeat retrotransposons in miniature (TRIMs), elongated non-coding derivatives and families retaining fragmented coding remnants. The identified families span a broad range, including elements exceeding 15,000 bp in length and display evidence for reshuffling and modular evolution. Only a subset of families could be confidently linked to autonomous retrotransposons, showing sequence diversification within the non-autonomous LTR retrotransposon fraction beyond the autonomous genomic templates.

We highlight that a large fraction of non-autonomous LTR retrotransposons is incompletely recovered with the current TE identification workflows, even if the output is well-curated and condensed into TE libraries and suggest procedures to remedy this gap. This study gives a genome-wide view into the non-autonomous LTR retrotransposon landscape of a single plant genome and highlights the importance of structure-based approaches for their identification and classification.

**Supplementary Information:**

The online version contains supplementary material available at https://doi.org/10.1186/s13100-026-00416-w.

## Introduction

Transposable elements (TEs) occupy large fractions of plant genomes. They accumulate in the genome [1–3], impact genomic structure and integrity [4, 5], and may cause genomic reorganization [6–8]. Based on their structural hallmarks, order of mobility-allowing protein domains, and the resulting movement processes, TEs are classified into multiple groups.

Here, we focus on long terminal repeat (LTR) retrotransposons, a large TE group propagating via a copy-and-paste mechanism with the help of an RNA intermediate [9]. Despite their variety, the overall structure is highly conserved and delimited by two identical LTRs (a result of the copy-and-paste mechanism). LTRs often contain signals for transcription initiation and termination [10–12]. The internal region between the LTRs contains binding sites for reverse transcription (the *primer binding site*; PBS and the *polypurine tract*; PPT) and protein domains for reverse transcription and integration. Usually, LTR retrotransposons are condensed into families based on the order and similarity of the coding domains [13].

Nevertheless, a variety of short LTR retrotransposons without reminiscence to the coding regions can also be identified. Similar to their autonomous counterparts, non-autonomous LTR retrotransposons are characterized by identical flanking LTRs and by the PBS and PPT regions. Non-autonomous LTR retrotransposons are typically shorter than their autonomous counterparts and often structurally diverse, including solo-LTRs, and tandemly-arranged and reshuffled copies [14–17]. Traditionally, these non-autonomous LTR retrotransposons have been subdivided based on their overall length: whereas *terminal-repeat retrotransposons in miniature* (TRIMs) [18] are described as short (< 1 kb), *large retrotransposon derivatives* (LARDs) [14] are long (> 4 kb). For the purpose of this manuscript, we will use TRIM as a unified designation for all non-autonomous, non-coding LTR retrotransposons, regardless of their length.

Non-autonomous LTR retrotransposons are difficult to identify in genome assemblies. Their low complexity and short length make them unsuitable targets for the current TE identification and classification approaches, which are typically either similarity- or signature- (structure-) based, or a combination of both [19–21]. We identify three hurdles in TRIM identification:

1. Similarity-dependent TE tools incompletely recover non-autonomous LTR retrotransposons: These tools search for known TE protein domains in genome assemblies. This is possible as coding regions show high conservation in nucleotide/amino acid composition across species [22–24]. As non-autonomous LTR retrotransposons do not encode protein domains, homology-based approaches either fail to identify them or classify them as fragmented derivatives of autonomous elements.
2. *De novo* identification produces many false-positives: As similarity-based methods fail, only *de novo* identification is possible, combined with signature-based approaches. Signature-based identification scans the genome for coding domains and conserved motifs, such as PBS sequences, combined with an all-by-all sequence comparison to identify repeated regions such as LTR sequences. Several signature-based tools for LTR *de novo* identification are available [25–27] with LTR_Finder [28] being the most known and best-performing (despite being slow [22]). The advantage of these tools is their ability to identify LTR retrotransposons not only based on the presence of coding or conserved motifs, but by the presence of identical flanking regions in the form of LTR sequences. This process still needs manual curation as LTR sequences as the only structural hallmark of these elements leads to many false-positive hits, especially as truncated or tandemly-arranged copies are also detected [29]. Especially in the case of non-autonomous LTR retrotransposons, manual curation is often waived and the hits discarded. As a result, these retrotransposons are often not included in TE databases.
3. Manual curation is impeded due to vague TRIM boundaries: The correct annotation of full-length retrotransposons depends on accurately determining the TE’s boundaries. This is difficult for non-autonomous elements. Self-comparison of sequences is an efficient strategy for boundary detection. Dotplots are useful to compare sequences [30], allowing detection and annotation of complex repeat types and modular similarity [20, 31–33]. Furthermore, dotplots illustrate sequence information in a way, that balances human- and machine-readability at the same time. This allows manual curation as well as image processing with one output.

In summary, despite their potential to contribute to genome regulation and structural variation, non-autonomous LTR retrotransposons are still barely investigated. Their limited sequence complexity is at fault, leading to an under-annotation in genome sequences. The TE community is aware that this annotation gap exists, but it is unclear whether it is large or negligible. We aim to evaluate the size of this blind spot with a use case focusing on a single plant genome, but with in-depth manual curation of every retained sequence: Specifically, we identify, manually verify and characterize non-autonomous LTR retrotransposons in a moderately-sized plant genome and evaluate how their in- or exclusion may impact studies that address genome regulation and structural variation.

As a genomic reference for our study, we chose the genome sequence of sugar beet (*B. vulgaris*) as its TE landscape is very well-understood (summarized in [8, 34]), with detailed studies focusing on different LTR retrotransposons [35–37] and non-autonomous elements [17, 38]. It is moderately-sized (758 Mb) with a high-quality long read-based genome assembly [39].

Here, we apply a semi-automated, signature-guided *de novo* identification strategy for non-autonomous LTR retrotransposons. By combining homology-based quantification and manual curation, we aim to retrieve a comprehensive dataset to assess their diversity, structural organization and genomic distribution. Our sugar beet use case focuses on non-autonomous LTR retrotransposons and thus enables a deeper look into their variety and impact of these often neglected and underestimated TEs.

## Materials and methods

### Overview of the identification workflow

**Fig. 1:**
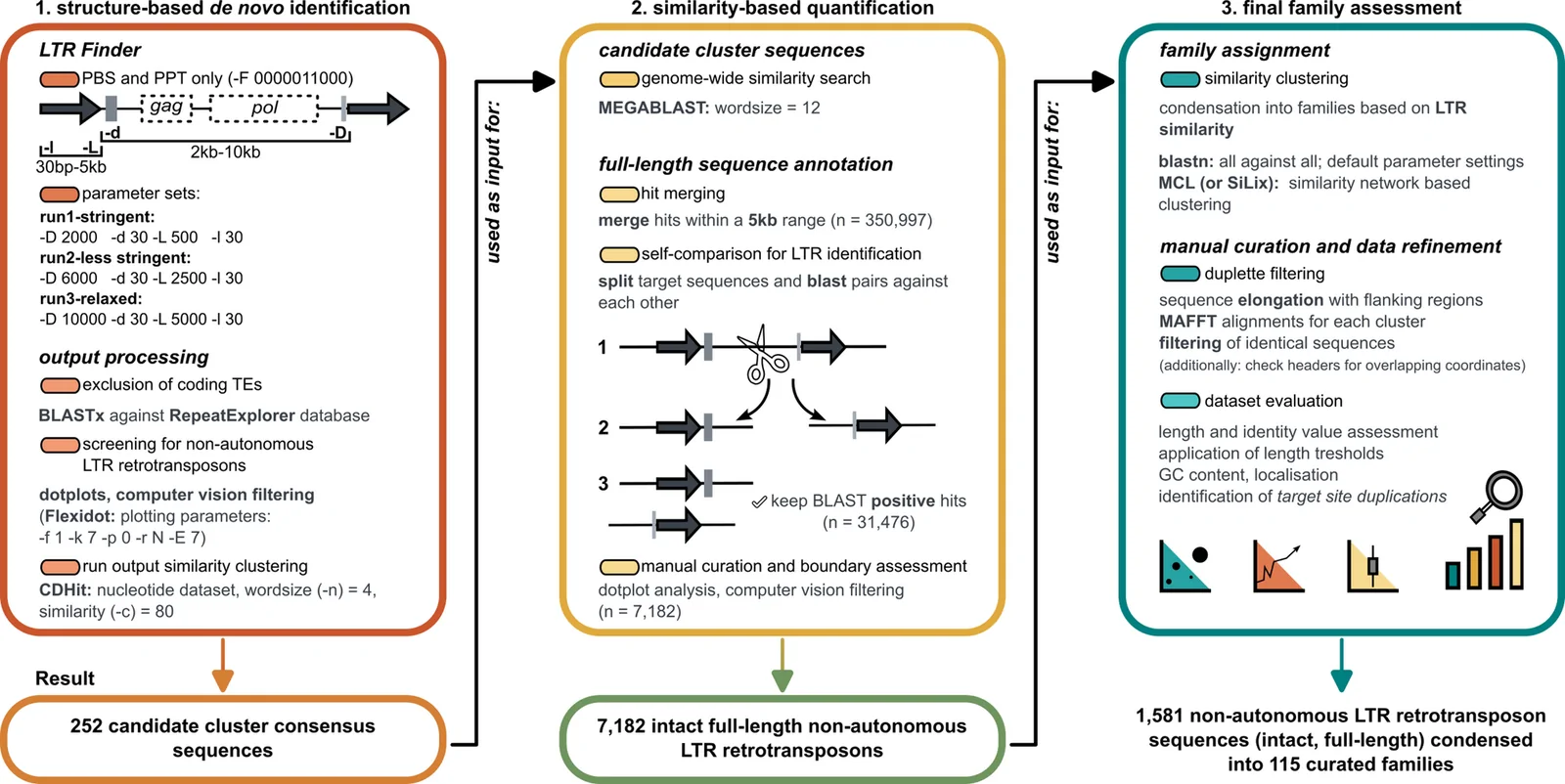
Three steps to identify non-autonomous LTR retrotransposons. As *input* we used an oxford nanopore technologies (ONT)-based genome assembly of *Beta vulgaris*. *Step 1* – Structure-based *de novo* identification and output processing; *Step 2* – Similarity-based quantification and annotation of full-length sequences; *Step 3* - Final family assessment via clustering and metadata evaluation

The workflow was designed to identify structurally intact non-autonomous LTR retrotransposons without relying on any conserved retrotransposon coding domains. Candidate elements were identified based of an LTR-like sequence architecture, followed by structural filtering, genome-wide quantification, clustering into families, and manual curation (Fig. 1).

The final dataset includes canonical TRIMs, elongated non-coding derivatives, and families retaining fragmented coding remnants, provided that no intact autonomous coding capacity was detectable (see TE list in Additional Table 1).

### *de novo* identification of candidate non-autonomous LTR retrotransposons

#### Initial LTR_finder screening

For the initial identification of non-autonomous LTR retrotransposons, we performed LTR_Finder analysis. As non-autonomous LTR retroelements frequently lack protein domains, we disabled protein domain detection and focused on structural LTR retrotransposon hallmarks. To maximize recovery of diverged elements, canonical 5′-TG…CA-3′ terminal motifs were not enforced during initial screening. Instead, motif detection was limited to primer binding site (PBS) and polypurine tract (PPT) prediction (-F 0000011000). PBS motifs were derived from the plant tRNA database [40].

We tested different parameter settings. As input, we used the Oxford Nanopore Technologies based genome assembly of sugar beet, *B. vulgaris* (GCA_947631125 [39]).

 To explore the whole size range of non-autonomous LTR retrotransposons three parameter sets with increasing relaxation of length constraints were evaluated to recover both miniature and elongated derivatives (Table 1).

**Table 1:** Parameter settings used for LTR_Finder analysis. LTR_Finder runs are sorted by stringency for short non-autonomous LTR-retrotransposons

Parameter settings	stringent*	less stringent	relaxed
min. LTR distance [ bp]	30	30	30
max. LTR distance [ bp]	2,000	6,000	10,000
min. LTR length [ bp]	30	30	30
max LTR lengths [ bp]	500	2,500	5,000

*Parameters suggested by Gao et al. 2016 [17]

#### Structural filtering and annotation of candidate elements

Candidate sets from all runs were combined and redundant reference sequences removed. Exclusion of autonomous LTR retrotransposon candidates was performed with BLASTx against protein-coding domains from the REXdb Viridiplantae v3.0 database (http://repeatexplorer.org/?page_id=918 [12]). However, downstream homology-based quantification occasionally recovered related copies containing coding remnants.

To reduce false-positive hits, we assessed the structural intactness of non-autonomous LTR retrotransposon candidates by plotting self-dotplots with Flexidot v1.06 [41]. Because of the shorter length and high variability, smaller word sizes are more suitable. Therefore, we adjusted the Flexidot plotting outputs with -f 1 (pdf output), -k 7 (word size), -p 0 (plotting mode: self), -r N (reverse complementary match plotting off), -E 7 (label size). For faster processing of dotplots we used a computer vision-based approach to search for LTR retrotransposon-specific patterns, laid out in Additional Fig. 1. Briefly, Flexidot outputs are split and binarized using Otsu thresholding [42], followed by contour detection and size filtering. For noise reduction, we used the morphological operation opening and a subsequent dilation with a 5 × 5 kernel. Additionally, largest contours for each plot are analyzed based on geometric properties (e.g. width, area, position) to distinguish structural arrangements. Sequences with no significant contours, especially those with fragmented repeat patterns or low-complexity noise, are filtered out.

#### Manual curation and structural annotation

All retained candidates were manually inspected in Geneious Prime. Candidates were retained when they displayed two recognizable terminal repeats, consistent internal organization, and absence of extensive structural disruption. Structural annotations included LTRs, PBS, PPT and internal sequence organization. PPT annotations were used as supportive evidence only and manually verified and corrected, if needed, as automated PPT prediction is often unspecific.

To reduce redundancy, candidate nucleotide sequences were clustered using CD-Hit [43] with a similarity threshold of 80% (-c 0.8), adapted to less conserved TEs. Representative sequences from each cluster (*n* = 252; Additional Fig. 2) were retained for genome-wide quantification. The sequences retrieved here will serve as a TE library.

### Genome-wide quantification and annotation of non-autonomous full-length LTR retrotransposons

#### Homology-based recovery of genomic copies

To reduce noise and increase yield, hits similar to the curated reference candidates were quantified via local MEGABLAST [44] against the KWS2320ONT assembly and a word size of 12. MEGABLAST was done with the open-source bioinformatics platform Galaxy (www.usegalaxy.org). The MEGABLAST output was converted to GFF format and hits within 5 kb were merged to enable assessment of tandemly-arranged copies. Extraction of FASTA sequences was performed with and without flanking regions up to 5 kb for identification of target site duplications (TSDs) inspection of nucleotide-level element boundaries and exclusion of false-positive structures, including highly similar satellite repeats.

#### Removal of fragmented and structurally incomplete copies

To enrich structurally intact elements and remove truncated fragments, solo-LTRs, and isolated local similarities, we applied a self-comparison filtering step.

For LTR retrieval, extracted hits were divided at their midpoints into artificial 5’ and 3’ sequence parts. These pairs were aligned against each other using BLAST-based self-comparison and all hits without positive self-comparison signals were removed. Sequences exceeding 10 kb were subdivided into quarters, as their length suggested a tandem arrangement. With further manual curation supported by multiple sequence alignments elements were classified as full-length if they presented intact PBS, consistent internal organization and intact LTR borders with 4nt terminal inverted repeats (TIRs). PPTs were used as supportive structural evidence as described above. Boundaries and flanking regions were additionally inspected to exclude false-positive structures, including pairs of highly similar satellite repeats.

### Clustering and family assignment

#### Cluster-based family assignment of intact full-length elements

As non-autonomous LTR retrotransposons frequently display length variation and modular internal organization, family assignment relied on LTR similarity rather than full-length multiple sequence alignments. For this, we tested:

- kmer-based identity evaluation:

We evaluated CD-Hit [45] and MMseqs2 [46] and with multiple parameter sets for each tool: (1) k-mer word size (CD-HIT: *n* = 4–9), (2) minimum sequence identity (CD-HIT: i = 80–90%; MMseqs2: i = 70–90%), and (3) minimum coverage/length overlap (CD-HIT: l = 70–90%; MMseqs2: l = 70–90%).

b.) Similarity-network based clustering:

Due to LTR sequence and length variation, alignment-based similarity calculations are unsuitable. We performed an all-against-all BLAST search (blastn; default parameters), followed by similarity-network clustering. We tested two graph-based clustering algorithms: SiLix [47] and MCL [48]. We chose pairwise similarity and alignment coverage thresholds for SiLix (identity: i = 70–90%, coverage: l = 70–90%) and explored inflation parameters (inflation: in = 2–5) for MCL.

Final clustering parameters were selected based on manual cluster inspection and family coherence. Due to modular organization of similarities occur across families, occasionally resulting in duplicated hits across clusters. Duplicated hits were excluded based on coordinates on the sugar beet pseudochromosomes and nucleotide composition including flanking regions. 

#### *Family recovery and length filtering after clustering*

Sequences differing more than 15% from median family lengths were manually excluded as indel-sequences. This removed highly truncated or insertion-containing variants that would otherwise inflate family length estimates. Full-length sequences representing the sole intact member of a family were retained following the same sequence-level structural validation applied to multi-copy families; homologous truncated and rearranged copies were assessed separately as additional family-level support.

#### Target site duplication (TSD) identification

TSD are indicators of genuine transposition events. For automated identification, we checked for identical motifs adjacent to annotated full-length element termini. We allow 1 mismatch, defining TSDs as 5 bp long sequence motifs with at least 80% identity.

#### Annotation of tandemly-arranged sequences and single LTRs

Tandemly-arranged sequences and single LTRs were identified with different strategies. For tandemly-arranged sequences, we scanned dotplots for TA-specific patterns. Candidate tandem structures were validated by BLAST comparison against family reference sequences. For single LTR annotation, we used the “solo_finder.pl” script from the LTR-retriever pipeline [49]. For both outputs, final annotation was performed manually.

#### Identification of truncated and re-arranged copies

To quantify structurally altered TRIM-derived sequences beyond canonical full-length elements, solo-LTRs and tandemly-arranged copies, all previously annotated full-length elements were removed from the MEGABLAST dataset. Remaining sequences, considered putative truncated and rearranged derivatives, were aligned against a curated non-autonomous reference dataset using BLAST (BLAST+ blastn, task blastn). BLAST search was performed with an increased maximum target sequence limit of 20,000 to avoid truncation of high-copy families.

BLAST alignments were filtered for ≥ 80% sequence identity, ≥ 100 bp alignment length, and ≥ 10% query coverage. Hits were grouped by reference sequence, and family-level statistics were calculated, including total hits, unique loci, unique contigs, mean alignment metrics, and the highest-scoring match. Unique loci were inferred from genomic coordinates encoded in subject sequence identifiers. This approach enabled genome-wide quantification of TRIM-derived sequences that retain detectable similarity to canonical family representatives but lack the structural features of intact elements, solo LTRs, or tandem arrangements, capturing truncated and rearranged derivatives.

#### Visualizations of gene association

Localization of TRIMs and autonomous LTR retrotransposons from sugar beet [34] was visualized with the circlize package in R [50]. We used the published sugar beet gene annotation [39] and overlapped it with LTR retrotransposons using BEDTools [51] for direct overlapping (bedtools intersect -a < TRIM dataset> -b < gene dataset> -wa -wb > < intersect output file> ) and associations within a 5 kb window in gene-flanking regions (extend genes by 5 kb each direction: bedtools slop -i < gene dataset> -g genome.sizes -l 5000 -r 5000 > < flank output file>; remove gene bodies: bedtools subtract -a < flank output file> -b < gene dataset> > < only flank output file>; intersect: bedtools intersect -a < only flank output file> -b < TRIM dataset> > < intersect with flank output> ).

For comparison with autonomous LTR retrotransposons, full-length Ty1-*copia* and Ty3-*gypsy* elements from the sugar beet repeat annotation [34] were extracted and analyzed using the same workflow. To avoid multiple counting of elements associated with overlapping gene models, gene-overlap and flank-overlap analyses were based on unique transposable element loci (bedtools intersect -u). The proportions of non-autonomous, Ty1-*copia*, and Ty3-*gypsy* elements overlapping genes or occurring within 5 kb of genes were compared using Fisher’s exact tests in R. Only elements located on pseudochromosomes were included in comparative gene-association analyses.

## Results

### Detection of non-autonomous elements on a genome-wide level

To investigate non-autonomous LTR retrotransposons two questions arise:

- A) “How many non-autonomous LTR retrotransposon families reside in the sugar beet genome?”; and B) “How effectively are these elements detected by current repeat identification approaches?”

We selected a plant genome, in which we retrieved and manually verified non-autonomous LTR retrotransposons. Here, we focus on the well-studied LTR retrotransposon landscape [17, 34–37, 52] of the moderately-sized sugar beet genome. Our semi-automated workflow consists of three steps (Fig. 1): (1) structure-based *de novo* candidate identification, (2) similarity-based genome-wide quantification and annotation of homologous copies and (3) final family assessment through clustering and manual curation.

#### Structure-based *de novo* identification

LTR_Finder with three parameter combinations recovered candidates with distinct length distributions reflecting the selected parameters (Additional Fig. 2A). Candidate numbers ranged from 6,021 in the stringent run to 39,143 in the relaxed run (Additional Fig. 2C). The majority of candidate sequences displayed low LTR_Finder scores, consistent with the absence of conserved coding domains in non-autonomous elements (Additional Fig. 2B). Exclusion of LTR retrotransposon coding domains reduced the dataset from 6,021 to 39,143 candidates to 1,737 − 27,687 structurally non-autonomous candidates, depending on the parameter set (Additional Fig. 2C).

Our computer vision-assisted filtering approach allowed rapid identification of sequences displaying characteristic LTR retrotransposon structures while removing candidates lacking clear paired terminal repeats. Rejected candidates included truncated, deleted or otherwise mutated hits, simple tandem repeats, nested transposable element fragments and sequences dominated by low-complexity regions. Clustering with CD-Hit and manual curation reduced the candidate set for stringent, less stringent and relaxed parameter runs to 136, 322 and 333 candidate sequences respectively (Additional Fig. 2C). After intersection of candidates and removal of redundant hits, 252 non-redundant reference sequences were retained for genome-wide quantification.

The 252 curated reference sequences were subsequently used for genome-wide homology searches.

#### Similarity-based quantification and annotation

The objective of this step was not to identify additional reference families, but to recover genomic copies belonging to the curated elements. MEGABLAST searches yielded 350,997 candidate loci throughout the sugar beet genome. To reduce noise such as solo-LTRs, fragmented artifacts and truncated copies, we checked each hit for presence of LTRs. This was done by mid-point splitting, followed by self-comparison as done by the DANTE-LTR pipeline [24]. Sequences lacking detectable terminal similarity were removed reducing candidate loci from 350,997 to 31,476. Subsequent inspection of self-dotplots, manual curation and assessment of intact LTR boundaries further reduced the dataset to 7,182 intact full-length non-autonomous LTR retrotransposons. During this final annotation step, PBS and PPT motifs were used as supportive structural evidence, while canonical TG...CA termini and major structural disruptions within LTR regions were evaluated manually.

#### Final family assessment

The resulting set of 7,182 intact non-autonomous full-length LTR retrotransposons was grouped into families. Clustering was performed based on LTR sequence similarity, as the most conserved family-specific feature.

Several clustering approaches and parameter combinations were evaluated (Additional Fig. 3). Among these, all-against-all BLAST followed by Markov Clustering Algorithm (MCL, -i(nflation) = 3; Additional Fig. 3D) yielded cluster numbers most consistent with manual curation. In contrast, SiLiX and MMSeqs2-based approaches split coherent families into multiple smaller clusters or singletons. All kmer based approaches resulted in many single-sequence clusters.

The final dataset of unique non-autonomous LTR retrotransposons after manual curationand applying strict length thresholds comprises 1,581 full-length sequences in 115 families with up to 95 intact full-length copies/family. The reduction from 7,182 to 1,581 reflects removal of duplicated loci, redundant family representatives, and structurally related variants during family-level curation.

We compared our curated dataset against the outputs of LTR_Finder and EDTA. Using default settings that include coding-domain detection, LTR_Finder recovered 86 of the 115 curated families. EDTA recovered sequence similarity corresponding to 97 families. However, these elements were classified broadly as “LTR/unknown” rather than distinct non-autonomous LTR retrotransposon families. Consequently, structurally intact non-autonomous elements will be combined with fragmented, recombined, and otherwise rearranged LTR retrotransposon derivatives. This will require extensive manual curation for family-level annotation.

### The non-autonomous LTR retrotransposon population is highly diverse: 42-fold difference in length and varying motifs

**Fig. 2:**
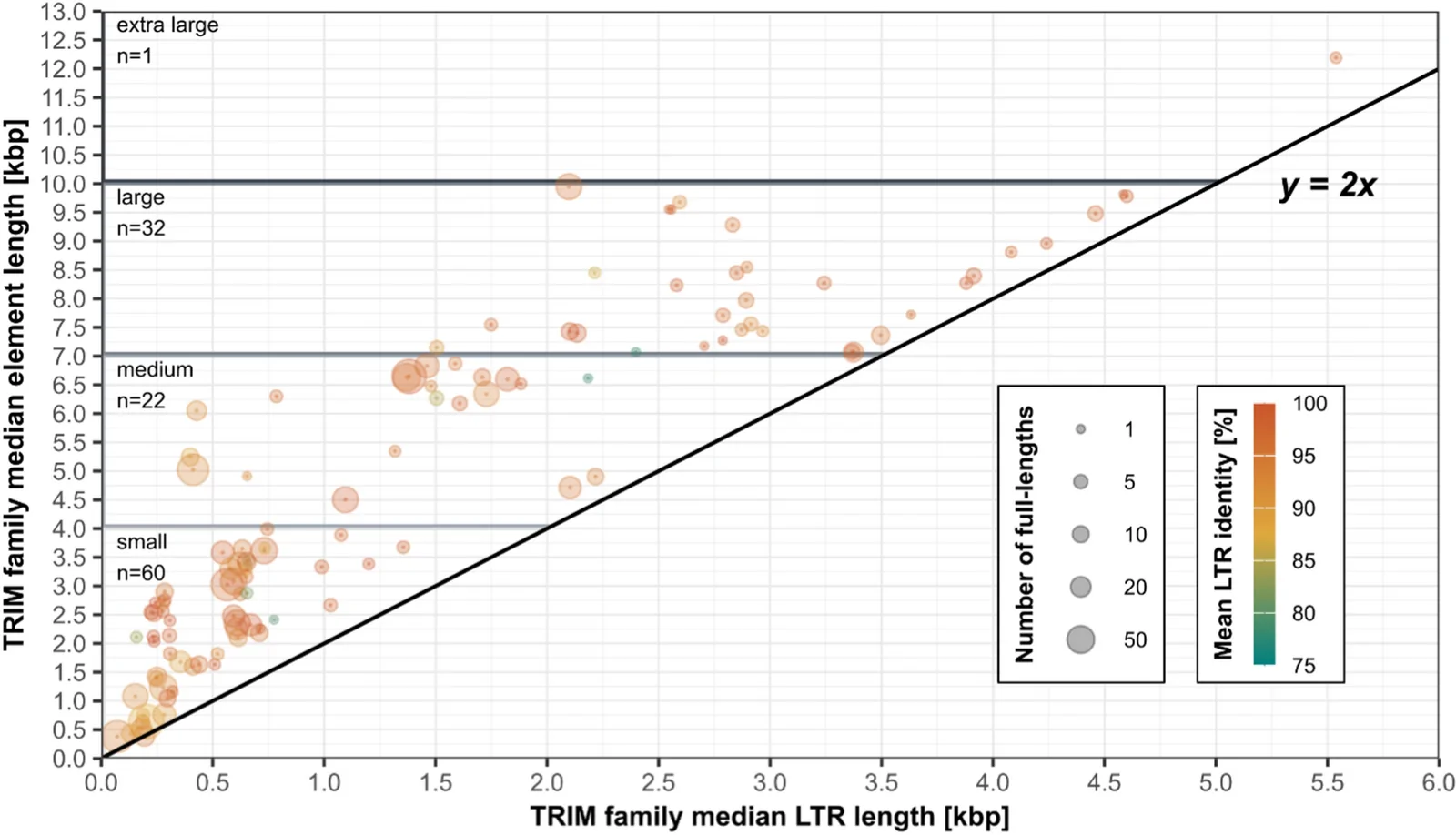
Length distributions across non-autonomous elements in sugar beet. Shown are all median LTR and corresponding full-length values for all identified families. Dots are scaled according to the number of full-length sequences representing each family. Colors indicate mean pairwise LTR identity values. The black line (y = 2x) refers to LTR and full-length combinations, arithmetically impossible to reach as they are smaller than the doubled LTR length

We examined structural diversity between the 1,581 curated non-autonomous LTR retrotransposons. Overall lengths ranged from 291 bp to 12.3 kb, representing a more than 40-fold size difference (Fig. 2). Although the majority of families exhibit a median length of less than 4 kb, we grouped the families into four categories according to median element length (Fig. 2): short (0–4 kb; *n* = 60), medium (> 4–7 kb; *n* = 22), long (> 7–10 kb; *n* = 32) and extra long (> 10 kb; *n* = 1).

No consistent relationship was observed between element size and LTR lengths (Fig. 2). Families with similar total lengths frequently differ in LTR size and internal region composition, indicating multiple structural configurations. Consequently, larger elements do not necessarily correspond to longer LTRs. For example, TRIM-06 has an overall length of 5.5 kb and LTR lengths of 186 bp, resulting in a length/LTR ratio of almost 15 (Additional Table 1.1).

Seventeen families have length/LTR ratios higher than 3.5, indicating long internal regions. Ten families (TRIM-03, -04, -11, -12, -14, -16, -19, -20, -33, and -34) contain internal satellite-like repeat arrays that contribute to expansion of internal regions (Additional Fig. 4). In contrast, 65 families exhibit ratios of 2 and less, reflecting short internal regions. A general tendency is visible: shorter internal regions are more common among families with greater overall length. This is particularly evident in families with LTR lengths of at least 2.1 kb, where ratios fall to 1.75 and less (Additional Table 1.1).

We observed high median pairwise LTR comparison values across the population. The majority of families show LTR identity values of 85% or higher (Fig. 2, color gradient), indicating homogeneous family populations. Lower median LTR identity values were observed in only five families (TRIM-04, TRIM-56, TRIM-58, TRIM-64 and TRIM-107). Notably, all five are represented by two or fewer full-length copies, suggesting that reduced identity values are primarily associated with small family sizes.

**Fig. 3:**
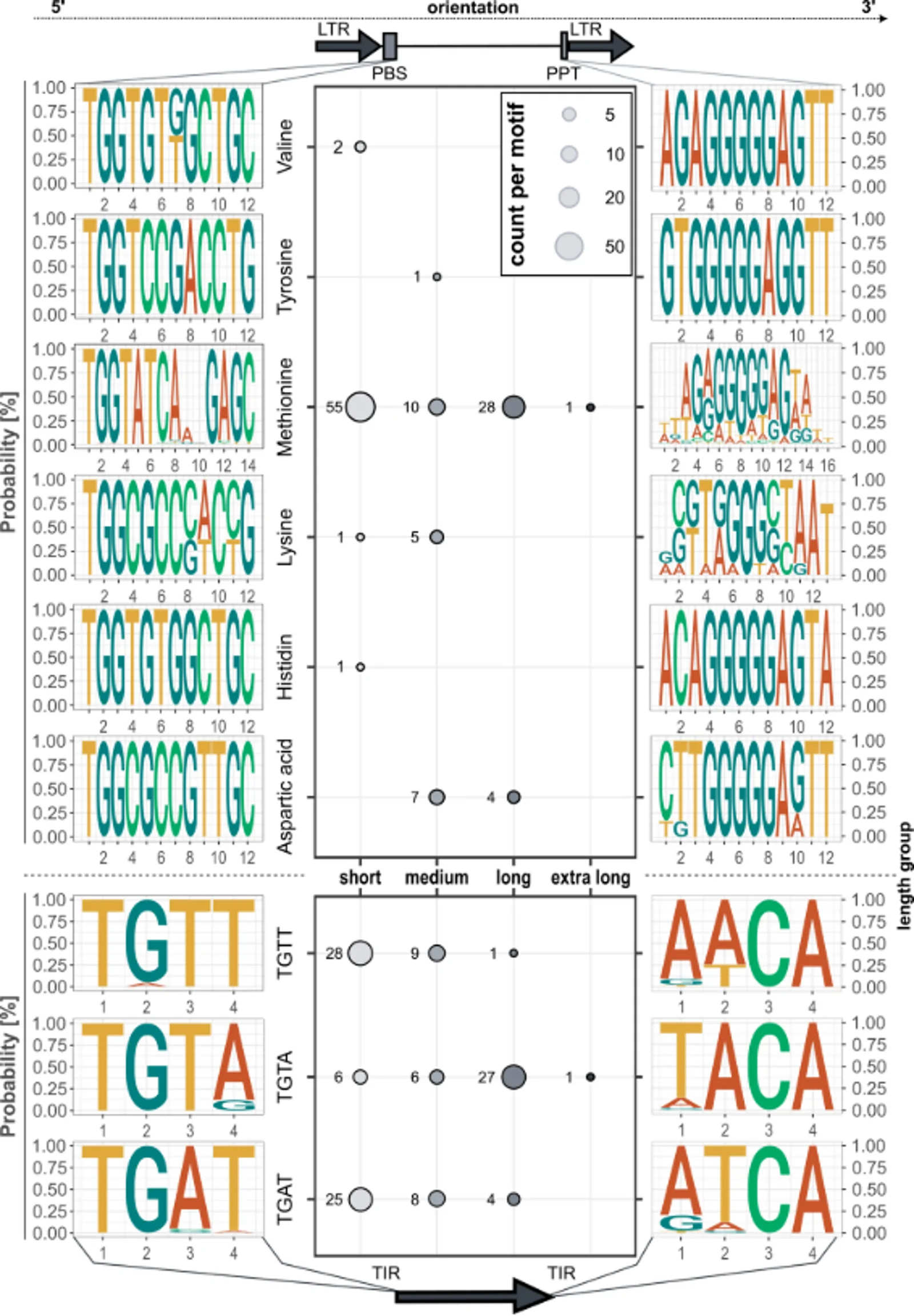
Analysis of identified primer binding site (PBS) motifs and the corresponding tRNA, polypurine tracts (PPT) and terminal inverted repeats (TIR). All identified tRNA-derived and TIR motifs and their corresponding 3’ PPT motifs as well as terminal inverted repeat (TIR) motifs are shown as sequence logos. Bubble sizes indicate the number of families carrying a given motif within each length class

Although non-coding, non-autonomous elements retain conserved motifs required for reverse transcription, such as the primer binding site (PBS). As PBS motifs are derived from specific tRNAs, they may provide clues to the evolutionary origin of individual families. In total, we identified six PBS sequence motifs across all 115 families (Fig. 3). Methionine-derived PBS motifs were the most common and occur across the entire length spectrum. Lysine and aspartic acid-derived PBS sequences are present in 6 and 11 families, respectively, mainly in medium-sized families. Other PBS motifs (histidine, tyrosine, and valine tRNA-derived) are less common and occur in one to two families. No obvious association between PBS type and family abundance was observed. In contrast to PBS motifs, PPT annotation did not reveal consistent family specificity (Fig. 3). Consequently, PPTs were treated as supportive structural features, but not for family assignments.

Finally, we analyzed the terminal motifs of the LTR (terminal inverted repeats; TIRs) associated with the conserved TG…CA LTR boundaries. We identified three major 4 bp TIR motif sequences across the defined length groups. 5’-TGTT-3’ and 5’-TGAT-3’ are most common in short families, while 5’-TGTA-3’ is dominant, but not exclusively present in longer families. Terminal motif composition showed only limited association with element size.

### Target site duplications and LTR conservation indicate recent integration

**Fig. 4:**
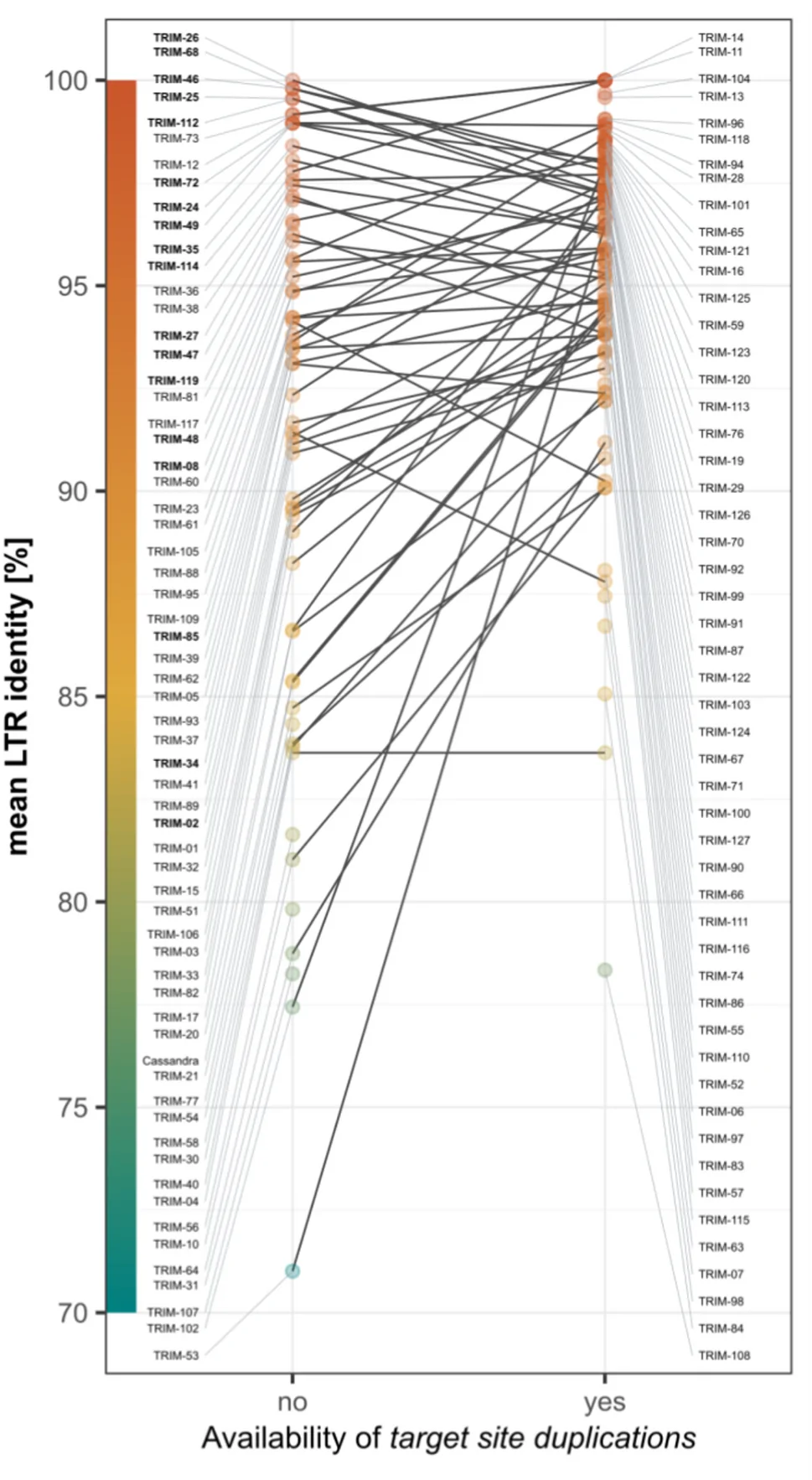
Comparison of *target site duplication* (TSD) availability and mean LTR identities per family. For each non-autonomous LTR retrotransposon family, the members with and without TSDs are separately listed. On the Y-axis, their LTR identity is shown. Higher identity values are shaded in orange. Labelled in bold are families with higher mean identity values for full-length copies without TSDs compared to those with

To assess structural conservation, we compared family-level mean LTR identities and the presence of target site duplications (TSDs; Fig. 4). These measures are more reliable than conventional substitution-based age calculations due to repeat homogenization and species-specific substitution rates [53, 54]. Across the dataset, most families displayed high LTR conservation. 79 families showed mean LTR identities of 95% or higher, while only two families exhibited mean LTR identities below 80% (Fig. 4).

Across all 1,581 full-length elements, 1,352 retained identifiable TSDs, whereas only 229 lacked them. At the family level, 108 of 115 families contain at least some TSD-positive full-length elements, demonstrating that TSD retention is common.

A relationship between LTR conservation and TSD retention was apparent. Elements from families with lower LTR identities (< 85%) most often lack TSDs, supporting the idea that older elements accumulate mutations in both their LTRs and flanking regions leading to the erosion of recognizable TSDs over time. However, this trend was not universal. Eleven highly conserved families with mean LTR similarities of > = 95% lack recognizable TSD motifs. These exceptions indicate that loss of TSDs cannot be explained solely by sequence divergence and is likely an indicator of genomic rearrangements and recombination.

Five families consist exclusively of TSD-negative elements (Additional Table 1.1). These families comprised only seven full-length copies. Consequently, complete absence of TSDs appears restricted to a small fraction of the population.

Overall, most families retain highly similar LTRs and recognizable TSDs, with exceptions indicating that recombination shaped them after integration.

### Recombination is common in non-autonomous LTR retrotransposon biology

To explore the impact of, we analyzed tandemly-arranged (TA) elements, solo-LTRs, chimeric elements and truncated or rearranged derivatives.

#### Tandemly-arranged copies are widespread

In total, we identified 97 TA copies across 38 families. Tandem structures ranged from 3 up to 21 LTRs. Most TA copies (*n* = 65) display a 3-LTR-structure. 15 families contain only a single TA copy, whereas the remaining families exhibit multiple tandem formations. TSD retention was observed in 73 TA copies, indicating that tandem formation occurs without the loss of insertion-associated hallmarks. High-copy families (e.g. TRIM-01) and single-copy families (TRIM-65, TRIM-125) contain tandem arrangements. Likewise, TA copies occur in families with high and moderate LTR identity.

#### Solo-LTRs occur in most families

In total, 101 of 115 families show evidence of solo-LTRs, while 14 families lacked them.

The abundance of solo-LTRs varied among families (Additional Table 1.1). Ratios of solo-LTRs to full-length elements range from 0.01 to 111. For instance, TRIM-01 contains 77 full-length copies, but only a single solo-LTR, whereas TRIM-26 contains 223 solo-LTRs but only two canonical full-length copies. Among the families with solo-LTRs, 54 have at least 50% of their solo-LTRs flanked by TSDs. Of these, 20 families contain TA copies and 18 exhibit TSD retention rates of at least 50% in TA copies and solo-LTR sequences.

#### Shared similarities and chimeric elements link families

In total, 55 families share regional sequence similarity and were condensed into 16 “modular groups” (M1-M16), visualized by dotplots (Additional Fig. 5).

Shared similarity and re-shuffling also occur in TA copies, showing sequence innovation across modular groups (e.g. M6 and M7). Re-shuffling and chimeric element formation also occur outside of modular groups, e.g. two chimeric elements assignable to TRIM-08 with additional LTRs donated by either TRIM-01 or TRIM-05 copies.

#### Truncated and rearranged derivatives are ubiquitous

In addition to canonical full-length elements, TA copies, and solo-LTRs, we detected evidence for truncated and rearranged elements in the sugar beet genome. In total 108 families retain detectable derivatives.

Across families, the derived fragments retained approximately 20% to 40% of the full-length copies. Despite this fragmentation, they often occur in large numbers. Families of modular group M16 (TRIM-120–126) illustrate this pattern particularly well: although their derivative sequences retain only 24–31% of the corresponding full-length representative, these fragments occur at hundreds of genomic loci. The prevalence of truncated or rearranged copies is not restricted to families with high abundance of TA copies or solo-LTRs. Families with few or no tandem arrays (e.g. TRIM-65) as well as those with higher tandem formation (e.g. TRIM-01 and TRIM-33) retained large numbers of derivative sequences.

### Not all non-autonomous LTR retrotransposons have a traceable origin

The evolutionary origin of non-autonomous families can be determined in two ways: (A) through the presence of residual protein domains derived from autonomous elements, and (B) through detectable sequence similarity to extant autonomous partners.

(A) Among the 115 families in our dataset, 43 retain remnants of coding domains from autonomous LTR retrotransposons as identified with DANTE [24]. These families comprised 10 Ty1-*copia* and 33 Ty3-*gypsy* related families. Based on their domain content (Additional Table 1.1 and 2), they fall into two categories.
  The first category consists of 28 families retaining only GAG/PROT domains, similar to TR-GAGs in *Coffea* [55], Arabidopsis and Triticaceae [56, 57].
  The second group comprises 15 families retaining at least one additional, partly decayed POL-associated domain, such as reverse transcriptase (RT), RNAseH (RH), Integrase (INT) and chromodomains (CHD). Eight retained GAG/PROT remnants, whereas seven lack them.
  Notably, 24 of 43 domain-carrying families show no detectable similarity to recent autonomous LTR retrotransposon in our sugar beet specific database [34]. In eight families, every identified copy retains coding remnants; all eight are small families with five or fewer full-length elements. More often, domain preservation is patchy: some family members retain coding fragments, whereas others display the non-autonomous structure with no remaining coding capacity (Additional Table 2).
(B) A second line of evidence was obtained from direct sequence similarity between non-autonomous elements and autonomous LTR retrotransposons. It is often assumed these elements establish a partnership with a corresponding autonomous element, enabling mobilization. Shared sequence similarity between autonomous and non-autonomous retrotransposons is often suggested to direct these partnerships and may enables allocation of non-autonomous families to either the Ty1-*copia* or the Ty3-*gypsy* superclade.
  In our dataset, 30 non-autonomous families could be linked to autonomous LTR retrotransposons in the sugar beet genome. These relationships can be grouped into three levels of similarity:

i.) high LTR similarity of non-autonomous/autonomous LTRs of 70% or higher (*n* = 10);
ii.) moderate LTR similarity (50–70%) between non-autonomous and autonomous elements (*n* = 12); and
iii) low LTR similarity (<50%), supported by similarity within internal regions, shared 5′ UTRs or retention of coding-domain fragments (*n* = 8).

Taken together, we determined the evolutionary origin for 54 of the 115 families. Twenty-four families were supported exclusively by coding-domain remnants, eleven exclusively by sequence similarity to autonomous sugar beet retrotransposons, and nineteen by both lines of evidence. Consequently, 61 families could not be linked to an autonomous partner using the currently available sugar beet genome resources.

For families retaining coding remnants, we additionally searched for microduplications that might mark breakpoints associated with loss of coding capacity. No consistent signal was detected. Instead, non-autonomous elements frequently contain short duplicated sequences within their internal regions (Additional Fig. 4), obscuring breakpoint signatures of individual deletion events.

Across all traceable non-autonomous families, the distribution between currently traceable sugar beet superfamilies is skewed: Ty1-*copia* (12 families) versus Ty3-*gypsy* (42 families), corresponding to an approximate ratio of 1 : 3.5.

### Non-autonomous LTR retrotransposons are scattered across the genome

**Fig. 5:**
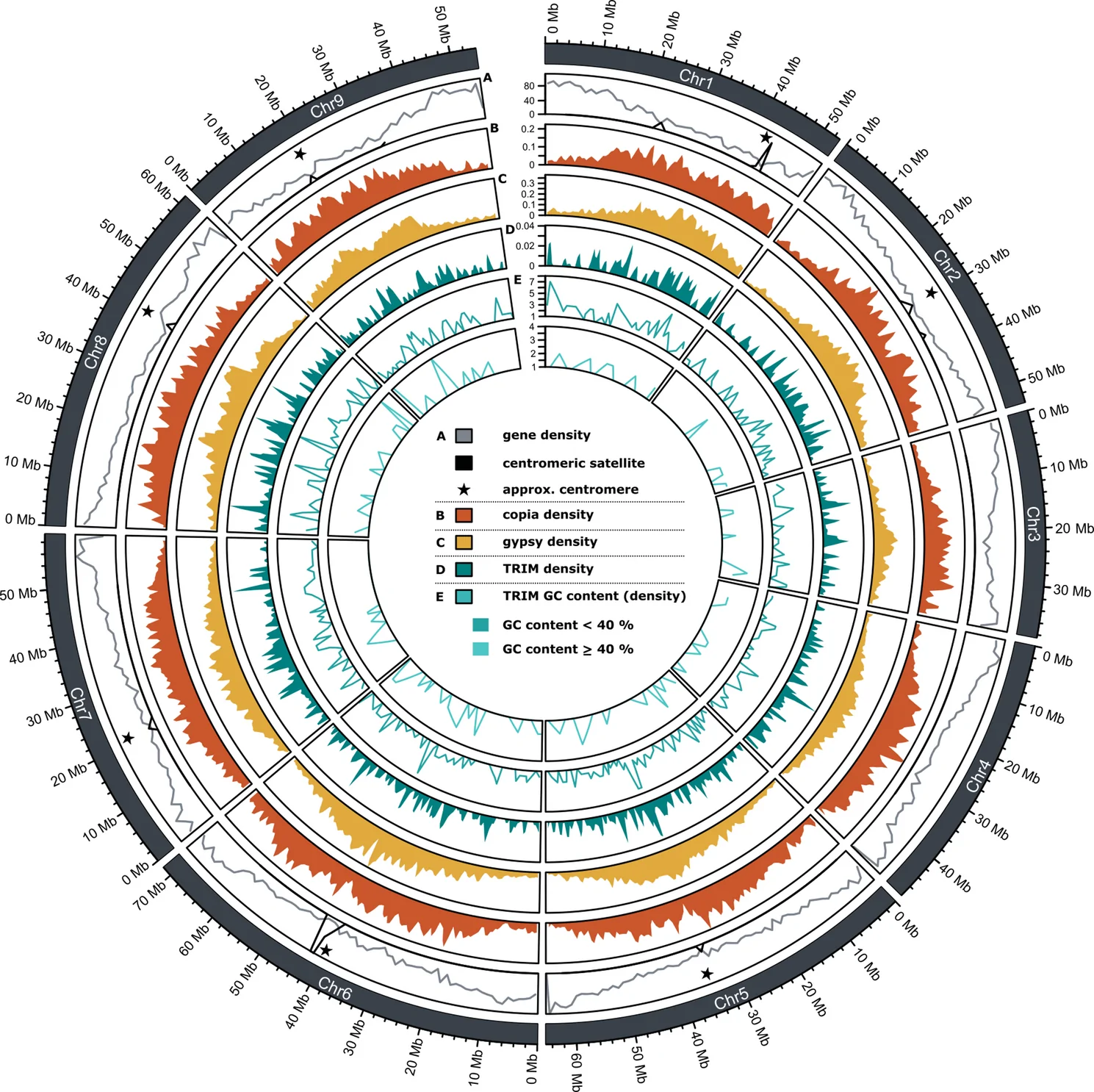
Localization of non-autonomous LTR retrotransposon full-length sequences across sugar beet pseudochromosomes. The density of autonomous LTR retrotransposons as well as non-autonomous LTR retrotransposons are shown as density plots with a window size of 1 Mb and are colored according to the superfamily (orange = Ty1-*copia*, yellow = Ty3-*gypsy*, blue = non-autonomous LTR retrotransposons/TRIMs). We furthermore added a track for the GC content of all full-length sequences within our dataset, which are shown in a lighter blue and are divided into two tracks for sequences with less or more than 40% GC. Centromeric regions are highlighted with asterisks and characterized by the abundance of centromeric satellite repeats

We analyzed the chromosomal positions of the full-length TRIMs along the assembled pseudochromosomes of the sugar beet reference genome (Fig. 5). Non-autonomous LTR retrotransposons showed a different chromosomal distribution from autonomous elements, with reduced density in centromeric regions (Fig. 5). Instead, non-autonomous elements reside in gene- and Ty1-*copia*-rich regions, especially along chromosome arms. The GC content of all full-length non-autonomous LTR retrotransposon sequences, ranged from 23% to 53%. Families with elevated GC content occur more frequently in gene-dense chromosomal regions, whereas GC-deprived elements showed a broader genomic distribution.

We compared the full-length dataset against the published gene annotation [39]. In total, 216 full-length elements overlapped annotated genes. Among these, 156 TE insertions (72%) occurred within introns, whereas 60 overlapped exons (Additional Table 3). An additional 627 non-autonomous LTR retrotransposons were located within 5 kb flanking regions. These gene proximal elements were distributed evenly between upstream (*n* = 315; 51%) and downstream (*n* = 312; 49%) positions, when analyzed in a strand-aware manner (Additional Table 3B). Individual copy insertions were associated with multiple neighboring genes (Additional Table 3 A), reflecting the high gene density of many chromosome-arm regions.

We compared gene associations of full-length non-autonomous elements with autonomous Ty1-*copia* and Ty3-*gypsy* elements. Gene-body overlaps were detected for 15.9% of non-autonomous elements, compared with 11.8% and 11.7% for Ty1-*copia* and Ty3-*gypsy* elements, respectively. Likewise, 39.8% of non-autonomous elements occurred within 5 kb of annotated genes, compared with 34.3% and 34.5% for autonomous Ty1-*copia* and Ty3-*gypsy* elements. These differences were significant in all comparisons (Fisher’s exact test, *P* < 0.001), with odds ratios ranging from 1.26 to 1.42, indicating a stronger association of non-autonomous LTR retrotransposons with gene-rich genomic regions.

Together, these observations demonstrate that non-autonomous LTR retrotransposons are preferentially located outside centromeric repeat-rich regions and occur within or near genes.

## Discussion

### Insights from sugar beet: non-autonomous LTR retrotransposons are underestimated, with different origins, and often without sequence similarity to autonomous TEs

In our sugar beet use case, we annotated 1581 full-length non-autonomous LTR retrotransposons of this moderately-sized plant genome. We avoided strict limitations (neither restricted lengths nor typical motifs) and relied on a complex semi-automated and manually verified workflow. Due to this permissive yet strictly controlled approach, we are confident in our catalogue of full-length TE references.

Despite this, some limitations apply: Contrary to common practice but to maximize completeness, we retained 31 families with less than three full-length members. These families are more likely to be impacted by genome assembly quality. Also, by removing eroded copies from our database, we biased our reference elements towards conserved elements.

The 1581 full-length non-autonomous LTR retrotransposons from sugar beet were analyzed in-depth to understand current annotation gaps and limitations, their evolutionary history and impact on genome biology. Key insights are:

#### Non-autonomous LTR retrotransposon diversity in length and sequence is vastly underestimated

Only eight out of the 115 families meet the strict TRIM definition ( < = 1 kb) and resemble typical representatives such as Wukong [58], Cassandra [17, 32, 59–61] and TRIMs from *Brassica*, apple and rice [62, 63]. Extending the definition to 1.5 kb [16] captures 15 families. Non-autonomous elements exceeding 4 kb (LARDs [14, 64]) correspond to 57 medium, long and extra long element sizes. However, intermediate size ranges from 1.5 to 4 kb remain undefined, despite reports of “long-TRIMs” in rice with lengths of 1.6 and 5 kb [65]. Conclusively, non-autonomous LTR retrotransposons form a structural continuum rather than a collection of discrete size classes. Despite their length variability, most families (96%) have high pairwise LTR similarity ( > = 85%), TSDs and high structural conservation. This is consistent with reports of rapid and recent integration of non-autonomous LTR retrotransposons into other plant genomes [66–68]. High- and low-copy families have high LTR identities, indicating family-specific mobilization dynamics.

Variability in PBS and TIR motifs further supports diversity of origin and modularity, with canonical methionine-tRNA-derived PBS motifs being most prevalent. However, less common PBSs derived from aspartate, tyrosine, histidine, and valine tRNAs were also detected, although these are rarely found in autonomous plant LTR retrotransposons [12]. Across all non-autonomous LTR retrotransposons in sugar beet, TIR motifs neither clearly correspond to predicted superfamilies nor show an association with TE length, unlike patterns reported for some autonomous elements in other plant genomes [69].

The observed diversity in length and motif sequence supports that non-autonomous LTR retrotransposons originate repeatedly from multiple autonomous lineages rather than a single ancestral source.

#### Partnerships with autonomous LTR retrotransposons are difficult to reconstruct

Non-autonomous elements without (complete) coding capacity require the enzymatic machinery of autonomous elements. Such cross-mobilization is well established for other non-autonomous TEs, such as (Alu) SINEs [70, 71], MITEs [72], and endogenous retroviruses [73] and suggested for non-autonomous LTR retrotransposons [18]. For instance, cross-mobilization has been shown for retrozymes, which rely on the Ty3-*gypsy* reverse-transcription machinery through partial sequence similarity [74].

Non-autonomous and autonomous LTR retrotransposons share similar sequences, as observed for the RLG_Quinta/RLG_Cereba system in wheat [75] and the BARE-1/BARE-2 pair in barley [76]. Hence, we compiled evidence for sequence-dependent mobilization by identifying regions or protein-domain fragments shared with autonomous LTR retrotransposons. In sugar beet, 30 of the 115 families show clear similarity to known autonomous elements through shared LTRs, internal sequences, or both. These relationships span both Ty1-*copia* and Ty3-*gypsy*, supporting repeated and independent emergence from multiple autonomous lineages. Nevertheless, high LTR identities with autonomous elements are observable in only 10 families, emphasizing that many autonomous progenitors may no longer be present in the current reference genome and mobilization may occur in a more generalistic manner. Similarly, protein domain remnants were detected in 43 of 115 families, however domain presence appears patchily across family members. This is similar to structural variation amongst autonomous LTR retrotransposons in plants [56] and TR-GAG elements in *Coffea* [55] and *Arabidopsis* [57].

Mobilization without sequence identity to an autonomous element presents an alternative strategy. Recently, in yeast, LTR retrotransposons were mobilized without an integrase [77], suggesting that cross-activation of defective elements can lead to mobilization even with minimal shared sequence identity [56]. Our beet use case supports this, with 61 families lacking any detectable similarity to autonomous elements yet showing signatures of recent mobilization (retained high LTR identities and TSDs). Autonomous progenitors may have diverged beyond recognition, been lost from the reference genome, or persist in other sugar beet individuals. Alternatively, these families may rely on generalist mobilization, where active autonomous elements provide the machinery for *trans* interaction [6, 18, 76].

Altogether, our observations suggest that non-autonomous LTR retrotransposons are mobilized in a decentralized manner, using any available enzymatic machinery, regardless of any sequence similarities. Nevertheless, resolving the molecular basis of these partnerships will require experimental approaches and comparative analyses across multiple genomes and populations.

#### Tandemly-arranged copies, solo-LTRs and more: recombination as evolutionary force towards retrotransposon diversification

Our data demonstrate that non-autonomous elements are strongly influenced by recombination-driven processes. They display dynamic turnover, reflecting reduced structural constraints and reliance on few conserved motifs needed for host-dependent propagation. Two major recombination routes exist: (1) illegitimate recombination, which generates truncated elements [78, 79] and (2) unequal homologous recombination between LTRs of the same or distinct elements, producing solo-LTRs or tandemly-arranged copies [78]. These recombination events are common in plants [80, 81] and our dataset provides a quantitative view of both pathways:

We observe evidence for illegitimate recombination, such as truncated hits, observed indels, and modular re-shuffling (Additional Table 1). Template switching was evident in several families. The emerging hybrid elements illustrate how recombination can reshuffle structural components across families, generating novel combinations of LTRs and internal regions [9, 82]. This adds to reports of template switching in sugar beet [36] and across other organisms [11, 83–89].

We also detected indicators of unequal homologous recombination: Solo-LTRs are detected in 102 of 115 families, with solo/full-length ratios spanning four orders of magnitude, from nearly absent to more than 100 : 1. Solo-LTRs in families with high LTR identity values suggests that recombination happens continuously, even in young TE populations and is not restricted to older and abundant TE copies. As TSD retention rate for solo-LTRs is only 49%, solo-LTR formation is favored by inter-element unequal recombination.

Tandemly-arranged copies are widespread in the sugar beet genome, occurring in 38 families and spanning all length and LTR sizes. Arrangement in tandems is well-described for Cassandra elements and other TRIMs [15, 17, 18, 90]. Approximately 71% of elements in tandem retain TSDs, indicating that they originate from intra-element recombination. Although less represented, formation of TA copies from ectopic recombination also contributes to sugar beet genome plasticity [91]. Tandem formation occurs independently of copy number, LTR identity or element length, contradicting earlier observations from soybean where tandemly-arranged elements are enriched among LTR retrotransposons with larger LTRs [92]. Likewise, solo-LTR formation shows no clear relationship to size, copy number or identity values, aligning with LTR retrotransposon studies in conifers [53]. Our results indicate that recombination acts on a wide length spectrum and even very short elements, like TRIM-01, can participate in tandem formation.

So, as length, copy number and LTR identities are not driving recombination, what is? The size ratio of full-length/LTR has been proposed to facilitate tandem formation [93], which is weakly supported by our data: only nine families with ratios larger than five lack detectable TA copies.

Taken together, we conclude that non-autonomous elements do not represent a “dead end” of LTR retrotransposon evolution. Instead, recombination, both homologous and illegitimate, is a major driver of structural diversification. This contributes to the dynamics of repeat landscapes in sugar beet and shows that recombination is not merely a decay mechanism but also facilitates structural reinvention.

#### Non-autonomous LTR retrotransposons in sugar beet preferentially occupy gene-rich regions

Distribution of non-autonomous LTR retrotransposons on sugar beet pseudochromosomes reveals that these elements are depleted from centromeric and pericentromeric regions, where heterochromatin and Ty3-*gypsy* retrotransposons accumulate, and instead occur along chromosome arms in gene-rich regions, as has been reported for other plants [16, 62, 63].

Several factors may contribute to this distribution. The small size of many non-autonomous elements may increase their tolerance within gene-rich regions relative to larger autonomous retrotransposons [18], as smaller TEs may impose fewer structural and regulatory constraints on surrounding regions [94]. Compared with autonomous Ty1-*copia* and Ty3-*gypsy* elements in sugar beet, non-autonomous LTR retrotransposons show significantly higher frequencies of both gene-body overlapping and occurrence within gene-flanking regions. This may support preferential persistence within gene-rich chromosomal compartments.

GC contents of plants vary widely and are species-specific [95]. In sugar beet, GC-richer families occur more frequently in gene-dense regions, such as patterns observed for autonomous elements such as Tos17 in rice [96]. However, high-copy families occur across both GC-rich and GC-poor groups and among families with and without identifiable autonomous partners.

#### Non-autonomous LTR retrotransposons in sugar beet contribute to genomic innovation

Non-autonomous LTR retrotransposons are composed of few modules: LTRs required for transcription, replication-associated motifs such as PBS and PPT sequences, and remnants of coding domains inherited from autonomous progenitors. Our dataset demonstrates that these modules evolve semi-independently. Coding domains can be lost, while structurally intact elements persist, and recombination can reshuffle LTRs and internal regions without destroying structural element composition.

The occurrence of TA copies, chimeric elements, and modular groups demonstrates that recombination exchanges structural components among non-autonomous families. Similar mechanisms exist for autonomous LTR retrotransposons, where recombination and domain exchange contribute to the emergence of new lineages [83–85]. Structural compactness and formation of tandemly-arranged elements suggest a role in forming satellite DNA-like repeats, as reported for other plant TEs [97].

LTR regions may acquire new motifs, such as rDNA- or tRNA-derived regions, as reported for Cassandra and Helenus/Ajax elements [17, 32, 59, 98]. Likewise, LTRs may acquire promoters and transcription factor binding sites, which could alter gene expression [99]. Although we did not investigate regulatory activity directly, the frequent localization of non-autonomous elements within gene-rich regions places them in contexts, where such interactions are possible.

Our sugar beet dataset suggests that non-autonomous LTR retrotransposons are not merely passive remnants of autonomous retrotransposon decay. Instead, they represent structurally dynamic genomic components that diversify through recombination, module exchange, and sequence turnover. Their contribution to genome evolution may extend beyond transposition and include broader effects on genome restructuring and long-term repeat landscape dynamics.

### Recommendations and limitations when identifying non-autonomous LTR retrotransposons

Non-autonomous LTR retrotransposons are structurally minimalistic and highly variable, making them among the most difficult elements to identify in plant genomes. Their lack of conserved protein-coding domains, broad size spectrum, and lineage-specific sequence divergence mean that conventional *de novo* LTR retrotransposon pipelines frequently underrepresent them or classify them as fragmented autonomous elements. Consequently, the diversity and genomic impact of non-autonomous LTR retrotransposons remain underestimated.

A major conclusion from this study is that identification of non-autonomous LTR retrotransposons benefits from a combination of complementary approaches rather than the reliance on a single detection strategy. Here, we performed a three-step routine, relying on (1) structure-based *de novo* identification, (2) similarity-based annotation, and (3) family assessment. Regarding structure-based identification, our evaluation suggests that moderate relaxation of minimum LTR length and LTR distance parameters is sufficient and preferable to default settings. This illustrates why pipelines using default LTR_Finder settings (including EDTA) miss portions of the non-autonomous LTR retrotransposon landscape.

The most important recommendations are the use of high-quality long-read assemblies, relaxed structural search parameters, careful separation of autonomous and non-autonomous copies, self-comparison-based validation, and graph-based clustering supported by manual curation. Manual curation and multiple sequence alignments remain essential for reconstructing family structures, within non-autonomous LTR retrotransposon landscapes [19, 100–102].

### Solving the dilemma of classification: How to name a TRIM, LARD, TR-GAG?

Despite two decades of research, terminology for non-autonomous LTR retrotransposons remains fractured. “TRIMs”, “long-TRIMs”, “SMARTs”, “LARDs”, “TR-GAGs”, and “defective” LTR retrotransposons all describe subsets of non-autonomous retroelements, but the boundaries between these categories blur. Our sugar beet dataset illustrates why classification is problematic.

Size thresholds do not map biological reality. Originally described as “TRIMs” or “SMRTs”, small non-autonomous elements were defined as less than 1 kb [18, 103]. In our dataset this applies to only 8 out of 115 families. If we extend the definition to less than 1.5 kb as proposed in Gao et al., 2016 [16] the threshold would still eliminate more than 85% of identified families. Elements larger than 4 kb or so-called LARDs [14] encompass all elements of medium sizes and above in our dataset. But no terminology exists for 1.5–4 kb, yet this group contains many trustworthy non-autonomous families in the sugar beet genome. Thus, this size-based terminology represents the extremes of a continuum, not discrete types.

Coding-capacity categories are equally ambiguous. The discovery of TR-GAG elements [55] introduced a category of non-autonomous retroelements that retain a GAG ORF while lacking enzymes and are considered “semi-autonomous” [56]. Our dataset includes 29 families with GAG/PROT domains, but these exceed the proposed size spectrum. An additional 14 families retain degraded fragments of RT, RH, or INT but lack complete coding capacity for self-driven mobilization. Thus, domain remnants serve as clues of evolutionary history but not as robust classification anchors.

Only a fraction of non-autonomous elements has a traceable origin. 30 of 115 families share detectable similarity to known autonomous elements in sugar beet, and only 10 can be assigned with > 70% LTR identity. Absence of similarity is influenced by recombination-mediated remodeling or lineage extinction of the former autonomous progenitor. Consequently, origin cannot be reliably inferred for most non-autonomous elements, even though their high LTR identities and TSD retention rates demonstrate recent activity.

None of the detected features in our dataset (PBS, PPT, terminal inverted repeats) allows classification. Likewise, length thresholds fail to capture the observed diversity, and autonomous ancestry can only be reconstructed for a subset of families. Therefore, current terminology does not reflect non-autonomous LTR retrotransposon diversity.

We propose a conceptual shift: instead of “non-autonomous” equating “short and non-coding”, we advocate using the term “non-autonomous” for elements with non-functional transposition, regardless of size and presence of fragmented ORFs. Within this category, classical TRIMs, long-TRIMs, LARDs, TR-GAG elements, and partially degraded derivatives represent different evolutionary paths of the same broader biological category. Autonomous ancestry frequently becomes obscured through recombination, sequence divergence, and loss of the original partner element. Consequently, classification based solely on autonomous superfamily affiliation becomes impossible for a substantial fraction of non-autonomous families.

We therefore suggest that annotation frameworks, repeat databases, and TE identification pipelines should introduce non-autonomous LTR retrotransposons as a third annotation category for classification. This follows the classification logic used for other non-autonomous TE orders, such as SINEs and MITEs, which already are recognized as biologically meaningful categories despite originating from autonomous ancestors. We furthermore advocate keeping definitions such as TR-GAG, Cassandra, or retrozymes to describe elements with designated features in a lineage-like manner.

## Conclusion

Non-autonomous LTR retrotransposons are difficult to identify and hence often insufficiently annotated in reference genome assemblies. Therefore, variability and abundance of non-autonomous LTR retrotransposons in plant genomes remained largely obscure. Here, we employed a sugar beet case study to remedy this gap for one plant example. We show that the retrieved non-autonomous elements are highly diverse in length and sequence and emerged repeatedly from diverse autonomous progenitors, forming 115 families. We highlight that reference TE databases encompassing only autonomous TEs would miss the vast majority of the non-autonomous LTR retrotransposon population. Non-autonomous elements are not static remnants but evolutionary dynamic participants in shaping plant genome architecture.

We conclude by giving recommendation for the identification of non-autonomous LTR retrotransposons in plant genomes and advocate to unify the terminology surrounding these elusive TEs.

## Supplementary Information

Supplementary Material 1.

Supplementary Material 2.

## Data Availability

All identified non-autonomous full length sequences as well as corresponding solo-LTRs and tandemly-arranged copies are publicly available on Zenodo: https://doi.org/10.5281/zenodo.18174724. Reference sequences for each TRIM family are available in the BeetRepeats database ([34] https://zenodo.org/records/8255813). Furthermore, detailed workflow description as well as code for file processing is stored under github: https://github.com/TRIMpossible/TRIM_identification. We used the large language models ChatGPT and GPT5 to assist with coding. All scripts are provided on github. The classifier workflow to recognize full length copy patterns from flexidot output dotplots is available in github as well: https://github.com/tudipffmgt/Flexidot-Classifier.
